# Supplementary material for: Differences in size and number of embryonic type II neuroblast lineages correlate with divergent timing of central complex development between beetle and fly
Source: eLife. 2025 May 6;13:RP99717. doi: 10.7554/eLife.99717 (PMC12055003; doi:10.7554/eLife.99717)
Supplement: Figure 2—figure supplement 1—source data 1. [file elife-99717-fig2-figsupp1-data1.zip › Figure 2- figure supplement 1- gel images source data/figure2-figure supplement1c- source data 1 2.pptx]

## Slide 1
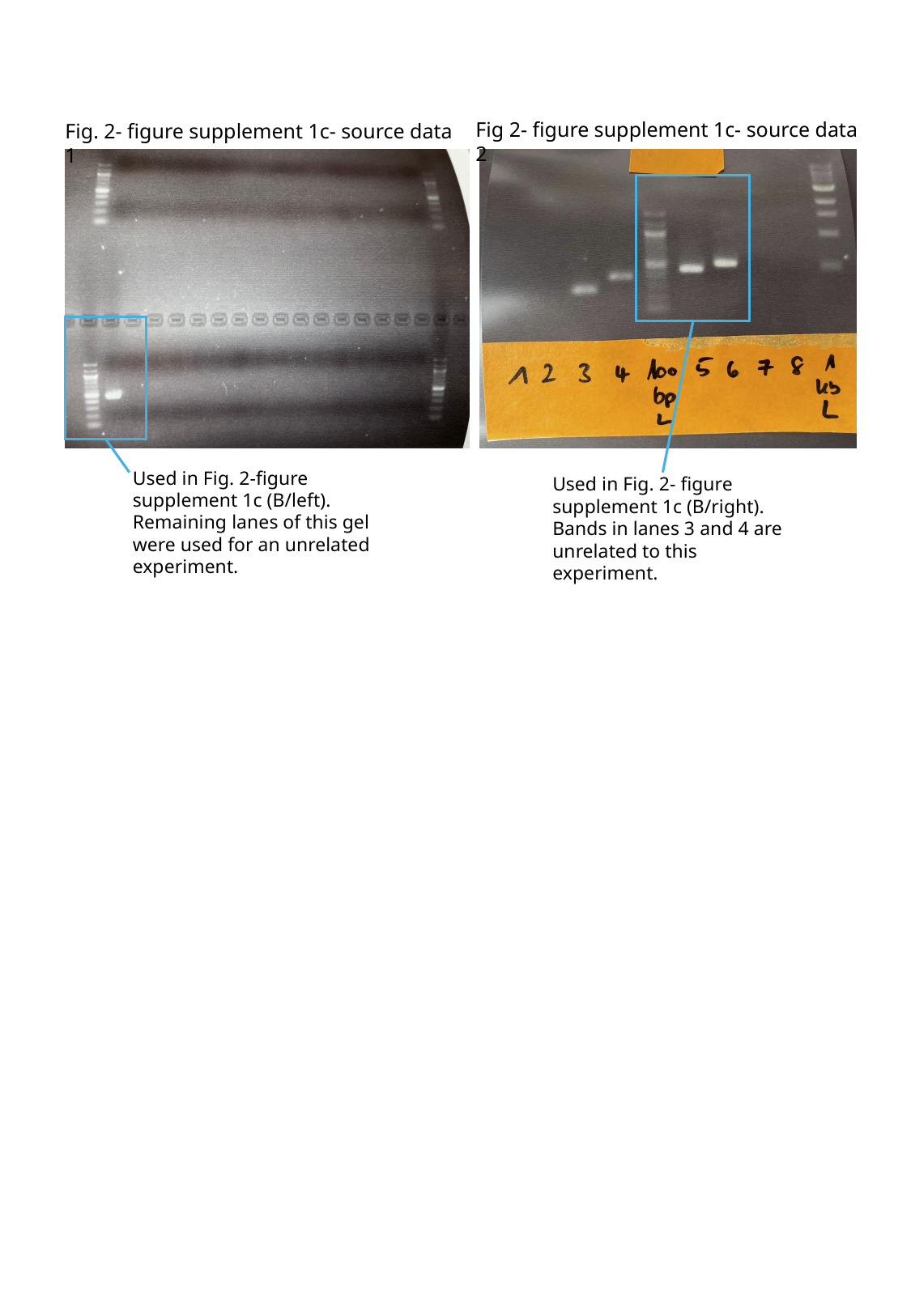

Fig 2- figure supplement 1c- source data 2
Fig. 2- figure supplement 1c- source data 1
Used in Fig. 2-figure supplement 1c (B/left). Remaining lanes of this gel were used for an unrelated experiment.
Used in Fig. 2- figure supplement 1c (B/right). Bands in lanes 3 and 4 are unrelated to this experiment.
