# Supplementary figures and images for: Differences in size and number of embryonic type II neuroblast lineages correlate with divergent timing of central complex development between beetle and fly
Source: eLife. 2025 May 6;13:RP99717. doi: 10.7554/eLife.99717 (PMC12055003; doi:10.7554/eLife.99717)

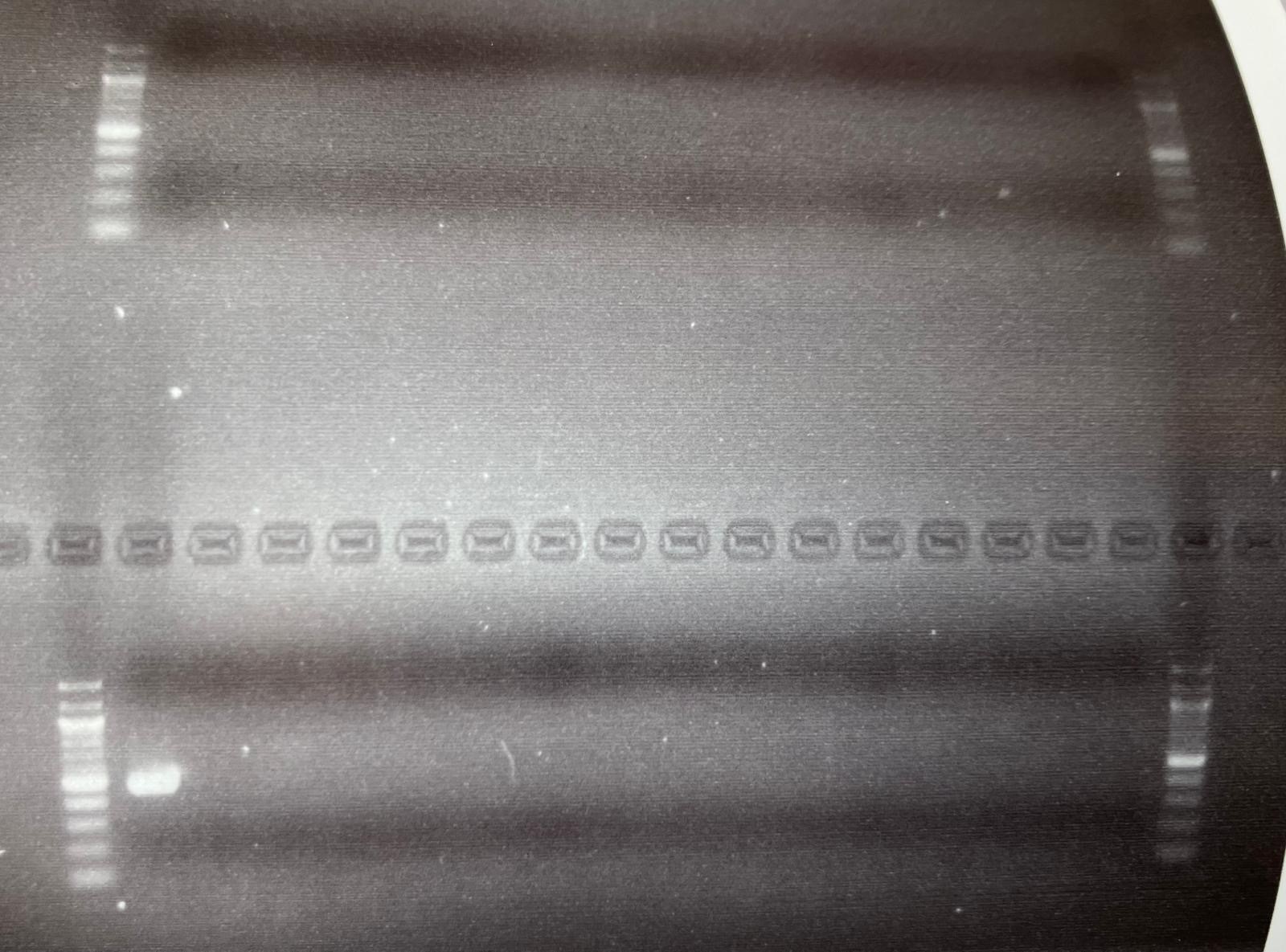

Supplement: Figure 2—figure supplement 1—source data 1. [file elife-99717-fig2-figsupp1-data1.zip › Figure 2- figure supplement 1- gel images source data/Figure2-figure supplement 1c-source data1.jpg]

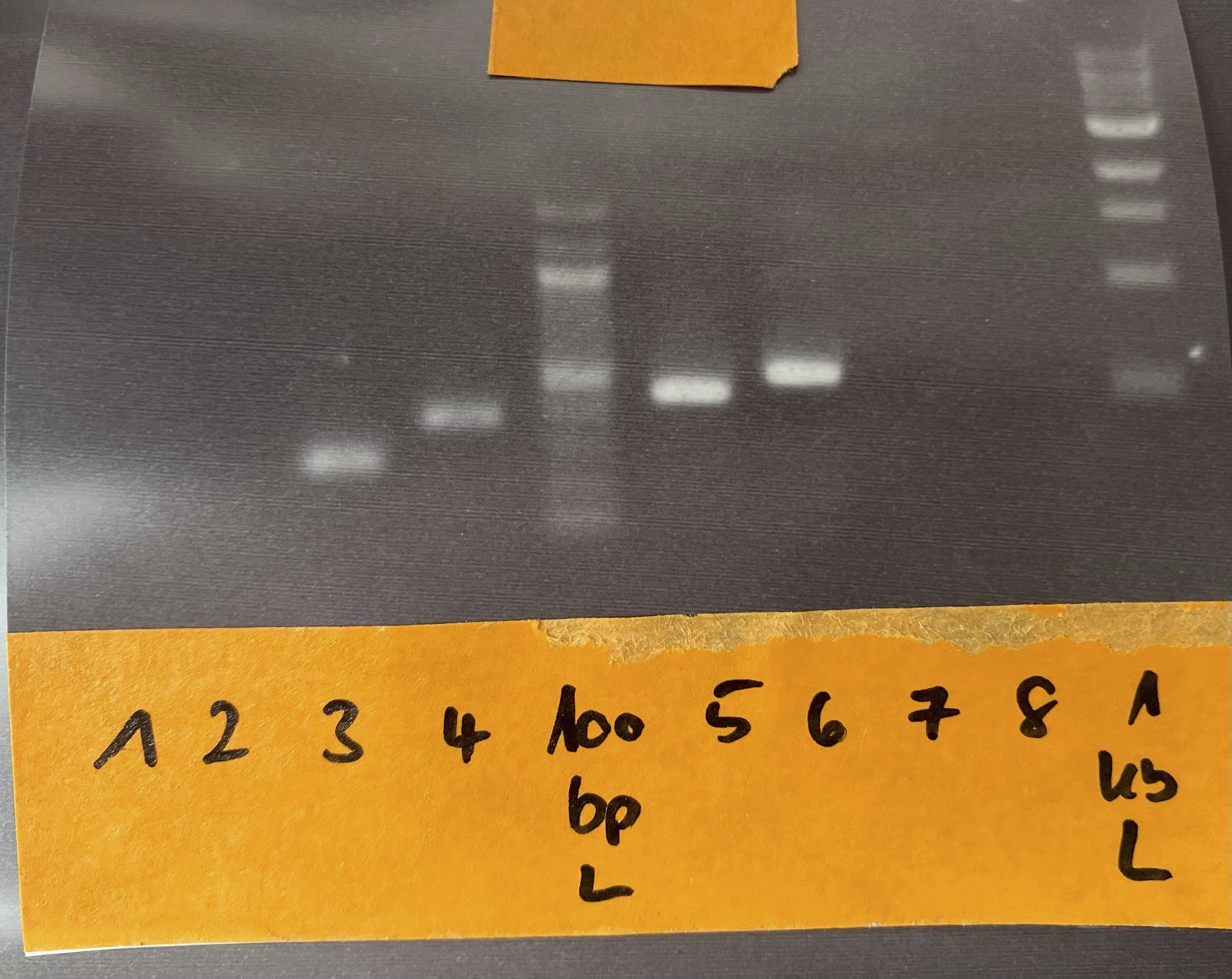

Supplement: Figure 2—figure supplement 1—source data 1. [file elife-99717-fig2-figsupp1-data1.zip › Figure 2- figure supplement 1- gel images source data/Figure2-figure supplement 1c-source data2.tiff]
