# Supplementary material for: Differences in size and number of embryonic type II neuroblast lineages correlate with divergent timing of central complex development between beetle and fly
Source: eLife. 2025 May 6;13:RP99717. doi: 10.7554/eLife.99717 (PMC12055003; doi:10.7554/eLife.99717)
Supplement: Supplementary file 1. — (a) Target sites for CRISPR-Cas9-mediated non-homologous end joining knock-in of the eGFP containing transgene (see Figure 2—figure supplement 1A). Guide sequences 1–3 are within 2.6 kb upstream of the transcription start site (TSS). (b) Primer sequences for line fez-mm-eGFP insertion site testing. See Figure 2—figure supplement 1A for primer binding sites. [file elife-99717-supp1.docx]

**Supplementary file 1a**

| CRISPR-guide RNA name | CRISPR-guide RNA sequence incl. PAM |
| --- | --- |
| *Tc-fez/erm* upstream 1 | GTGATTACGTGCCGCCGAAG TGG |
| *Tc-fez/erm* upstream 2 | GCGCTTGCTCGGTTCTCAGT TGG |
| *Tc-fez/erm* upstream 3 | GCCGTCGTGAGTGAAACGCC AGG |
| *Dm-ebony* | GAACCGGGCAGCCCGCCTCC TGG |
| *Dm-yellow* | GCGATATAGTTGGAGCCAGC TGG |

**Supplementary file 1b**

| Primer name | Primer sequence | Distance/ lane in Fig. 2- figure suppl. 1 (B) |
| --- | --- | --- |
| GFP-5'-rv1 | TGAACTTGTGGCCGTTTACG | 443 bp /1 |
| Fez-ex1-rv1 | AACATTAGGTGAGCAGGGCC |  |
| GFP-fw-1 | TTCTTCAAGGACGACGGCAA | 474 bp /2 |
| P2A-rv | TCTTCCACGTCTCCTGCTTG |  |
| GFP-fw-1 | see above | 543 bp /3 |
| Cre-rv1 | GTTGCATCGACCGGTAATGC |  |
